# Cost per patient of treatment for rifampicin‐resistant tuberculosis in a community‐based programme in Khayelitsha, South Africa

**DOI:** 10.1111/tmi.12544

**Published:** 2015-06-01

**Authors:** Helen Cox, Lebogang Ramma, Lynne Wilkinson, Virginia Azevedo, Edina Sinanovic

**Affiliations:** ^1^ Division of Medical Microbiology and Institute of Infectious Disease and Molecular MedicineUniversity of Cape TownCape TownSouth Africa; ^2^ Health Economics UnitUniversity of Cape TownCape TownSouth Africa; ^3^ Médecins Sans Frontières (MSF)KhayelitshaSouth Africa; ^4^ City of Cape Town Health DepartmentKhayelitshaSouth Africa

**Keywords:** multidrug‐resistant tuberculosis, rifampicin‐resistant tuberculosis, treatment, cost, decentralisation, South Africa, TB‐MDR, TB‐RR, traitement, coût, décentralisation, Afrique du Sud

## Abstract

**Objectives:**

The high cost of rifampicin‐resistant tuberculosis (RR‐TB) treatment hinders treatment access. South Africa has a high RR‐TB burden, and national policy outlines decentralisation to improve access and reduce costs. We analysed health system costs associated with RR‐TB treatment by drug resistance profile and treatment outcome in a decentralised programme.

**Methods:**

Retrospective, routinely collected patient‐level data were combined with unit cost data to determine costs for each patient in a cohort treated between January 2009 and December 2011. Drug costs were based on recommended regimens according to drug resistance and treatment duration. Hospitalisation costs were estimated based on admission/discharge dates, while clinic visit and diagnostic/monitoring costs were estimated according to recommendations and treatment duration. Missing data were imputed.

**Results:**

Among 467 patients (72% HIV infected), 49% were successfully treated. Treatment was initiated in primary care for 62%, with the remainder as inpatients. The mean cost per patient treated was $7916 (range 260–87 140), ranging from $5369 among patients who did not complete treatment to $23 006 for treatment failure. Mean cost for successful treatment was $8359 (2585–32 506). Second‐line drug resistance was associated with a mean cost of $15 567 *vs*. $6852 for only first‐line resistance, with the major cost difference due to hospitalisation. Costs are reported in 2013 USD.

**Conclusions:**

RR‐TB treatment cost was high and varied according to treatment outcome. Despite decentralisation, hospitalisation remained a significant cost, particularly among those with more extensive resistance and those with treatment failure. These cost estimates can be used to model the impact of new interventions to improve patient outcomes.

## Abstract (fr)

**Objectifs:**

Le coût élevé du traitement de la tuberculose résistante à la rifampicine (TB‐RR) empêche l'accès au traitement. L'Afrique du Sud subie une forte charge de TB‐RR et la politique nationale applique la décentralisation pour améliorer l'accès et réduire les coûts. Nous avons analysé les coûts du système de santé associés au traitement de la TB‐RR selon le profil de résistance aux médicaments et le résultat du traitement, dans un programme décentralisé.

**Méthodes:**

Les données rétrospectives à l’échelle du patient, systématiquement recueillies ont été combinées avec les données des coûts unitaires pour déterminer les coûts pour chaque patient dans une cohorte traitée entre janvier 2009 et décembre 2011. Les coûts des médicaments ont été basés sur les schémas recommandés selon la résistance aux médicaments et la durée de traitement. Les coûts d'hospitalisation ont été estimés sur base des dates d'admission/de sortie, alors que les coûts des visites à la clinique et du diagnostic/suivi ont été estimés selon les recommandations et la durée du traitement. Les données manquantes ont été prises en compte.

**Résultats:**

Sur 467 patients (72% infectés par le VIH), 49% ont été traités avec succès. Le traitement a été initié dans les soins primaires pour 62% et pour les autres en tant que patients hospitalisés. Le coût moyen par malade traité était de 7916$ (intervalle: 260–87 140); allant de 5369$ chez les patients qui n'ont pas terminé le traitement à 23 006 $ pour l’échec du traitement. Le coût moyen pour un traitement avec succès était de 8359 $ (2585–32 506). La résistance aux médicaments de 2nd ligne a été associée à un coût moyen de 15 567 $ par rapport à 6852 $ pour la résistance de première ligne seule, avec la plus importante différence de coût due à l'hospitalisation. Coûts estimés sur base de l’USD 2013.

**Conclusions:**

Le coût du traitement de la TB‐RR était élevé et varie en fonction de l'issue du traitement. Malgré la décentralisation, l'hospitalisation est restée une source de coût important, en particulier pour ceux avec une résistance plus étendue et ceux avec un échec de traitement. Ces estimations de coûts peuvent être utilisées pour modéliser l'impact de nouvelles interventions visant à améliorer les résultats des patients.

## Abstract (es)

**Objetivos:**

El alto coste del tratamiento de la tuberculosis resistente a la rifampicina (TB‐ RR) dificulta el acceso al tratamiento. Sudáfrica tiene una gran carga de TB‐RR, y las políticas nacionales hacen énfasis en la descentralización con el fin de mejorar el acceso y reducir los costes. Hemos analizado los costes del sistema sanitario asociados al tratamiento de la TB‐ RR, en un programa descentralizado, según el perfil de resistencia y el resultado del tratamiento.

**Métodos:**

De forma retrospectiva, combinamos datos rutinarios de pacientes con datos de coste unitarios para determinar los costes por paciente dentro de una cohorte tratada entre Enero del 2009 y Diciembre del 2011. Los costes de medicamentos se calcularon sobre los regimenes recomendados según los niveles de resistencia y la duración del tratamiento. Los costes de hospitalización se calcularon basándose en las fechas de admisión/alta, mientras que las visitas a la clínica y los costes de diagnóstico / monitorización se calcularon según las recomendaciones y la duración del tratamiento. Los datos faltantes fueron imputados.

**Resultados:**

De 467 pacientes (72% infectados con VIH), un 49% fueron tratados con éxito. El tratamiento se inició en atención primaria para un 62%, con el resto como pacientes hospitalizados. El coste medio por paciente tratado era de $7916 (rango 260–87 140); desde $5369 para pacientes que no completaban el tratamiento hasta $23 006 cuando había fallo en el tratamiento. El coste medio para un tratamiento exitoso era de $8359 (2585–32 506). La resistencia a la segunda línea de tratamiento estaba asociada con un coste medio de $15 567 versus $6852 para solo la primera línea de tratamiento, siendo el de la hospitalización la mayor diferencia en el coste. Los costes se reportaron en USD del 2013.

**Conclusiones:**

Los costes de tratamiento de la TB‐RR eran altos y variaban según el resultado del tratamiento. A pesar de la descentralización, la hospitalización seguía siendo un coste significativo, particularmente entre aquellos con resistencias más extensas y quienes presentaban fallo en el tratamiento. Los costes aquí calculados pueden utilizarse para modelar el impacto de nuevas intervenciones que mejoren los resultados en pacientes.

## Introduction

Tuberculosis drug resistance is now a major barrier to TB control globally. Despite this reality, only 20% of estimated multidrug‐resistant tuberculosis (MDR‐TB) cases receive appropriate second‐line treatment 1. The high cost of second‐line treatment for MDR‐TB, estimated at an average of USD 9235 in low‐income countries 1, is a significant barrier to treatment provision, despite estimates of cost‐effectiveness in several settings 2.

The high costs of treatment are primarily driven by the cost of second‐line drugs, and the model of care, that is the extent of hospitalisation 2. While previously, patients with MDR‐TB were often treated in specialist hospitals as inpatients, increasing patient numbers and the need to initiate treatment rapidly in order to reduce both mortality and ongoing transmission have led to recommendations for ambulatory models of care 3. Comprehensive decentralisation allows integration with existing routine drug‐susceptible TB services and HIV services, in high HIV prevalent settings, and provides a more patient‐centred approach 4. In addition to improving access to treatment, decentralisation may also reduce costs. South Africa has a high burden of MDR‐TB, with more than 26 000 cases of rifampicin‐resistant TB (RR‐TB) diagnosed in 2013 1. Despite this high burden, this represents a relatively small fraction of the overall TB burden, with fewer than 7% of positive Xpert MTB/RIF results nationally recorded as rifampicin resistant 5. Among diagnosed cases in 2013, only 10 663 (41%) patients were reported to have started second‐line TB treatment, and yet the cost of managing drug‐resistant TB was reported to account for a third of the total TB programme budget 1. WHO estimate that the 55 million USD currently allocated to RR‐TB needs to be increased to 336 million in order to fully respond to the TB epidemic and treat all diagnosed cases in South Africa 1.

Prior to 2011, South African guidelines recommended hospitalisation of all MDR‐TB patients for at least 6 months or until culture conversion. A study of costs during this period found a mean cost per patient treated over 12 months of USD 17 164, of which 95% was due to hospitalisation 6. In response to increasing patient numbers, long delays to access inpatient treatment and increasing costs, the South African guidelines were revised in 2011 to recommend hospitalisation only for sputum smear‐positive patients until conversion to negative (approximately 2 months) and for patients with extensively drug‐resistant TB (XDR‐TB) 7. Estimates of the cost of treating drug‐resistant TB under the new guidelines range from USD 6772 for MDR‐TB to USD 26 392 for XDR‐TB 8.

However, these estimates were based on costs calculated if all patients were successfully treated according to the revised treatment guidelines, and do not take into account differing treatment duration and hospital stay due to the adverse outcomes of death, early treatment discontinuation and treatment failure. In addition, the extent of ambulatory, decentralised care for RR‐TB, as recommended in the guidelines, varies across South Africa, and in several provinces, RR‐TB treatment remains highly centralised, provided through specialist TB hospitals. In order to provide more realistic cost data to inform policy, we aimed to conduct an analysis of health system costs associated with RR‐TB treatment, and relate costs to drug resistance profile and treatment outcomes, in a decentralised, community‐based programme in Khayelitsha, South Africa.

## Methods

### Study setting

This study was conducted in Khayelitsha, the largest township in the Western Cape Province of South Africa, with a population of approximately 400 000 people. Nearly 40% of the workforce is unemployed, and 55% of households are classified as informal dwellings 9. Khayelitsha has extremely high burdens of HIV, TB and DR‐TB, with antenatal HIV prevalence reported at 34% and TB case notification approximately 1200/100 000/year 10, 11. Rifampicin‐resistant TB was found among 5.2% of new and 11.1% of previously treated TB cases in a survey conducted in 2008/09 12. Approximately 200 patients are diagnosed with rifampicin‐resistant TB annually 13.

Khayelitsha has 11 healthcare facilities providing primary care; 8 primary care clinics and 3 larger community health centres (CHCs). The larger clinics and the CHCs have full‐time medical officer support, while others are attended by a medical officer once or twice a week. There is also a secondary level district hospital, in addition to a separate subacute inpatient facility specifically for drug‐resistant tuberculosis.

### RR‐TB treatment programme

The decentralised, community‐based RR‐TB programme in Khayelitsha has been previously described 13, 14. The programme was implemented collaboratively by Médecins sans Frontières (MSF), the City of Cape Town and the Provincial Government of the Western Cape. Implementation was incremental, starting with extensive training of primary care clinic staff and clinic‐based registers. Further inputs included the following: individual‐specific RR‐TB counselling, social assistance and support groups, routine home visits, clinician support, a local inpatient service and audiometry screening. By 2010, the majority of the programme was considered to be in place.

A key aspect of the decentralised programme is the provision of ambulatory RR‐TB treatment initiation and management by primary healthcare clinicians for patients able to attend their local primary care clinic daily. Patients requiring hospitalisation for their clinical condition are referred to the central TB hospital in Cape Town for admission and treatment initiation. The model also includes the provision of counselling both for patients at diagnosis and through treatment, and for families and household initially. Healthcare workers involved in RR‐TB care at primary care level are supported through initial and ongoing training sessions. Support for clinicians is provided through a monthly multidisciplinary patient review meeting in which all new patients initiating treatment as well as other complicated cases are reviewed. Specialist advice is available at this meeting as required.

As the specialist TB hospital in Cape Town is located some distance away from Khayelitsha, a local inpatient service was established. This is a 12‐bed inpatient facility, staffed by nurses, offering subacute care. The facility is used primarily for short‐term admission for patients requiring an intermediate level of clinical care, with continued responsibility and management by the clinic primary healthcare doctors.

### Study population

For the analysis of costs per patient, patients with bacteriologically confirmed RR‐TB started on treatment between January 2009 and December 2011 were included in the patient cohort. Patients with bacteriologically unconfirmed RR‐TB (primarily children) were excluded, as were subsequent treatment episodes for the same patient. Patients for whom the treatment outcome was recorded as transferred or not available and those with unknown treatment duration were also excluded. All data were compiled from a routinely maintained electronic database, with data entry from clinic registers and patient records as previously described 13.

Drug resistance categories included the following: rifampicin mono‐resistance (Rif‐mono, no demonstrated isoniazid resistance), MDR‐TB (resistance to rifampicin and isoniazid with no demonstrated second‐line resistance), pre‐XDR (FLQ) (MDR‐TB with resistance to a fluoroquinolone, generally ofloxacin), pre‐XDR (INJ) (MDR‐TB with resistance to a second‐line injectable drug, either kanamycin or capreomycin) and XDR‐TB (MDR‐TB with resistance to both a fluoroquinolone and a second‐line injectable drug). Treatment outcomes were defined according to South African guidelines 7.

### Cost analysis

Health system or provider costs were determined for each individual patient in the cohort using an ingredient's approach, that is multiplying unit costs (to the health system) by resource use. Costs for four categories were estimated for each patient: drug use, hospital stay, clinic use and diagnostic/monitoring test use. Data were collected in South African Rand (ZAR) and, where required, inflated to 2013 rates using the medical consumer price index of 6.4% for 2011 and 6.1% for 2012 15, 16. Data were converted to US dollars using the 2013 average annual exchange rate of US$1 = ZAR9.3 (OANDA Currency converter 2014, average exchange rate for January–December 2013. http://www.oanda.com).

#### Resource use

Drug use was extrapolated based on the treatment regimen recommended for each drug resistance profile category 7 (Table 1) multiplied by the durations of the intensive (receipt of the second‐line injectable drug) and continuation phases actually received by each patient (missing intensive phase durations were imputed). Hospital stay was estimated based on recorded admission and discharge dates (with imputation for missing data).

**Table 1:** Cost per day of drug regimens according to South African national guidelines by drug resistance category (in 2013 USD)

Drug resistance profile	Treatment regimen	Cost per day	
		Intensive phase	Continuation phase
RIF‐mono	Int: Kan/Mox/Tzd/Ethio/INH Cont: Mox/Tzd/Ethio/INH	3.64	3.20
MDR‐TB	Int: Kan/Mox/Tzd/Ethio Cont: Mox/Tzd/Ethio	3.64	3.20
Pre‐XDR (FLQ)	Int: Kan/Mox/Tzd/Ethio/PAS/Clof Cont: Mox/Tzd/Ethio/PAS/Clof	9.33	8.88
Pre‐XDR (INJ)	Int: Cap/Mox/Tzd/Ethio/PAS/Clof Cont: Mox/Tzd/Ethio/PAS/Clof	18.93	8.88
XDR‐TB	Int: Cap/Mox/Tzd/Ethio/PAS/Clof Cont: Mox/Tzd/Ethio/PAS/Clof	18.93	8.88

Int, intensive phase; Cont, continuation phase; RIF‐mono, rifampicin mono‐resistance; MDR‐TB, multidrug‐resistant TB; Pre‐XDR (FLQ), MDR‐TB plus fluoroquinolone resistance; Pre‐XDR (INJ), MDR‐TB plus second‐line injectable resistance; XDR‐TB, extensively drug‐resistant TB; Kan, kanamycin; Mox, moxifloxacin; Tzd, Terizidine; Ethio, ethionamide; INH, isoniazid; PAS, para‐amino salicylic acid; Clof, clofazimine.

Clinic visits with medical consultation were allocated at treatment initiation and monthly during ambulatory treatment, including admission to the subacute facility. Nurse‐led primary care clinic visits were allocated for 5 days/week during the intensive phase and weekly during the continuation phase. Total clinic use was estimated using the number of clinic visits and the ambulatory treatment duration (i.e. duration of treatment received in primary care). Similarly, diagnostic and monitoring test use was estimated according to recommended guidance (rather than actual) and multiplied by treatment duration.

The mean duration of intensive phase treatment was used to impute data for patients with missing intensive phase duration. If total treatment duration was less than the mean duration of the intensive phase, all treatment was assumed to be of intensive phase, that is including the injectable drug. Otherwise, the mean duration of intensive phase treatment for successfully treated patients was used for missing values. Similarly, for missing data on length of stay (LOS), the mean LOS by each treatment outcome and for admission to either the central TB hospital or the local RR‐TB inpatient facility was calculated and used to infer missing values. If the total treatment duration was less than that imputed, then total treatment duration was used as LOS for that patient.

#### Unit costs

Unit costs for RR‐TB medical consultation and nurse‐led primary care clinic visits were obtained from an earlier, related study and were averaged from 3 health facilities in Khayelitsha 17. Similarly, the cost per inpatient day in the central TB hospital was also obtained from the previous study, and the same methods were used to determine the cost per inpatient day in the RR‐TB subacute inpatient facility in the current study 17. Briefly, data were collected from a range of sources, including management records and interviews with staff, using an ingredients approach. Capital and overhead costs were included. Costs of laboratory‐based tests for diagnosis, further drug susceptibility testing (DST) and TB treatment monitoring tests were obtained from a wider study into the introduction of the rapid Xpert test in South Africa, the XTEND study [Cunnama L. et al. Unpublished data]. In this study, costs were determined using a bottom‐up approach based on the use of detailed records and observation of resource use. Unit costs from previously published South African studies were used for the cost of non‐TB baseline and monitoring tests 8. The cost of drugs was derived from the National central medical depot tender price list (www.doh.gov.za/mpc.php 2012). All unit costs are described in Table 2.

**Table 2:** Unit cost utilised and source (in 2013 USD)

Cost component	Unit cost (USD)	Source
Clinic visit (medical consultation)	10.88	(17)
Clinic visit (drug collection/injections)	4.89	(17)
Inpatient day (central TB Hospital)	44.44	(17)
Inpatient day (local sub‐acute facility)	117.62	Study data
Xpert MTB/RIF	16.9	Cunnama L. et al. Unpublished data
Microscopy tests	6.3	Cunnama L. et al. Unpublished data
Sputum liquid culture	12.9	Cunnama L. et al. Unpublished data
First‐line drug sensitivity test (LPA)	20.3	Cunnama L. et al. Unpublished data
Second‐line DST (2 drugs)	25.1	Cunnama L. et al. Unpublished data
X‐ray (digital)	24.01	South African Department of Home Affairs, 2013 public sector price
Kidney test	12.45	(8)
Liver functioning test	17.24	(8)
TSH	23.94	(8)
Audiogram	29.28	(8)
HIV rapid screening test	6.03	(8)
CD4 count + viral load	59.30	(8)

USD, US dollars; LPA, line probe assay; DST, drug susceptibility testing; TSH, thyroid stimulating hormone; HIV, human immunodeficiency virus.

### Ethics approval

The maintenance of a routine database and evaluation of the Khayelitsha RR‐TB programme were approved by the Human Research Ethics Committee at the University of Cape Town. Further approvals were sought for the collection of cost data for this study from the same committee.

## Results

### Study cohort

There were 550 culture‐confirmed RR‐TB patients started on treatment for the first time between January 2009 and December 2011; 13 patients were excluded from the final cohort due to missing treatment duration or missing treatment outcome. A further 70 patients were excluded with the outcome of transferred out. Among the final cohort of 467 patients, 72% were HIV infected, 48% were female, and the mean age was 33 years (range 1–71). Overall, 49% of patients were successfully treated. Treatment outcomes by drug resistance profile are described in Table 3.

**Table 3:** Treatment outcomes by drug resistance category for the study cohort

Drug resistance profile	Treatment outcomes				
	Treatment success (%)	Loss from treatment (%)	Treatment failure (%)	Died (%)	Total
R‐mono	55 (55)	35 (35)	1 (1)	10 (10)	101
MDR‐TB	158 (51)	99 (32)	10 (3)	42 (14)	309
Pre‐XDR (FLQ)	3 (27)	2 (18)	3 (27)	3 (27)	11
Pre‐XDR (INJ)	7 (27)	5 (19)	3 (12)	11 (42)	26
XDR‐TB	5 (25)	0 (0)	4 (20)	11 (55)	20
Total	228 (49)	141 (30)	21 (4)	77 (16)	467

RIF‐mono, rifampicin mono‐resistance; MDR‐TB, multidrug‐resistant TB; Pre‐XDR (FLQ), MDR‐TB plus fluoroquinolone resistance; Pre‐XDR (INJ), MDR‐TB plus second‐line injectable resistance; XDR‐TB, extensively drug‐resistant TB.

Total treatment duration varied according to treatment outcome, with longer mean duration for successfully treated patients and the shortest duration among patients who died during treatment (Table 4). Among successfully treated patients, the mean duration of the intensive phase was 194 days (Table 4). There were 101 patients with total treatment duration less than 194 days with missing intensive phase duration. For these patients, all treatment was presumed to be of intensive phase. There were 68 patients with total treatment duration longer than 194 days with missing intensive phase duration; intensive phase duration was assumed to be 194 days for these patients.

**Table 4:** Mean (SD, standard deviation) total and intensive phase treatment duration by treatment outcome

Treatment outcome	*N*	Total treatment duration (days) Mean (SD)	*N* (available data)	Intensive phase duration (days) Mean (SD)
Treatment success	228	716 (88)	214	194 (50)
Loss from treatment	141	266 (182)	114	144 (83)
Treatment failure	21	435 (159)	8	178 (39)
Died	77	190 (186)	64	102 (82)
Total	467	482 (275)	400	164 (75)

The majority of patients (62%, 291/467) were started on RR‐TB treatment in primary care, with a further 16% (74/467) admitted to the subacute facility to start treatment. The remaining 21% (99/467) were admitted to the central TB facility to initiate treatment, based primarily on clinical need, but also for extensive drug resistance for a smaller number of patients. While admission dates were recorded routinely, discharge dates were missing for 4% (3/74) of patients admitted to the subacute facility and 37% (37/99) patients admitted to the central TB hospital. Average length of stay (ALOS) was calculated by treatment outcome for patients admitted to hospital to initiate treatment (Table 5). Further hospital admission, during treatment, was required for 51 patients. LOS was known for 39 (76%) of these and imputed for the remainder‐based similarly on treatment outcome (Table 5).

**Table 5:** Admission and length of hospitalisation by treatment outcome

Treatment outcome	*N*	Admitted for treatment initiation (%)	Central TB hospital^a^		Sub‐acute facility		Admission during treatment^b^	
			*N* (known LOS)	ALOS (SD)	*N* (known LOS)	ALOS (SD)	*N* (known LOS)	ALOS (SD)
Treatment success	228	74 (32)	24	115 (58)	32	59 (66)	22	46 (46)
Loss from treatment	141	43 (30)	12	138 (40)	22	52 (63)	7	47 (41)
Treatment failure	21	7 (33)	*N* = 4^c^, ALOS = 313 days (163)				6	190 (245)
Died	77	52 (68)	23	73 (59)	19	71 (107)	4	86 (64)
Total	467	176 (38)	62	119 (84)	74	74 (86)	39	71 (113)

ALOS, average length of stay; SD, standard deviation.

a Includes patients admitted to other tertiary hospitals and transferred subsequently to the central TB hospital.

b Includes all inpatient facilities due to small numbers.

c Both hospitals were combined for treatment failure patients due to small numbers.

### Cost per patient

**Figure 1:**
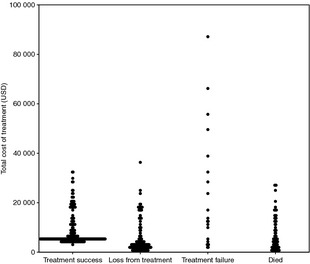
Scatter plot of total cost of treatment per patient by treatment outcome.

The mean total cost per patient treated was USD 7916, ranging from USD260 to USD 87 140 (Table 6 and Figure 1). This large variation resulted primarily from the variation in treatment duration, ranging from patients treated for just a few days before either dying or refusing further treatment to those treated for several years, most often in the case of treatment failure. Patients who did not complete treatment had the lowest mean and median cost at USD 5369 and 2732, respectively, and those for whom treatment failed, the highest mean cost at USD 23 006. While the mean cost among patients who were successfully treated was USD 8359, the overall cost per treatment success, taking into account all patients started on treatment, was USD 16 214.

**Table 6:** Mean, median and range of costs per patient by treatment outcome (In 2013 USD)

Treatment outcome	Mean cost (USD)	Median cost (USD)	Range
Treatment success	8359	5307	2585–32 506
Loss from treatment	5369	2732	260–36 319
Treatment failure	23 006	13 010	2203–87 140
Died	7151	4669	292–27 456
Total	7916	5054	260–87 140

Despite the majority of patients not being admitted to hospital for treatment initiation, hospitalisation remained the largest contributor to overall cost at a mean cost of USD 4100 per patient, with the highest cost of USD 77 276 accruing to the hospitalisation of a single patient with XDR‐TB for whom treatment failed (Table 7).

**Table 7:** Mean, median and range of costs per patient by cost area (in 2013 USD)

Cost area	Mean cost (USD)	Median cost (USD)	Range
Hospitalisation	4100	0	0–77 276
Drug regimen	2013	2122	4–9304
Primary health care	920	1003	11–2241
Diagnosis and treatment monitoring	883	952	221–1576

**Figure 2:**
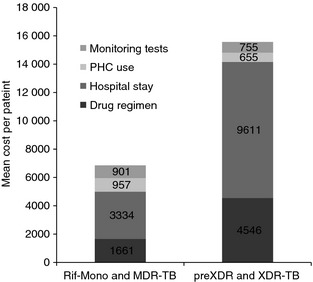
Comparison of mean cost‐by‐cost category between rifampicin mono‐resistant/MDR‐TB cases and MDR‐TB with second‐line resistance (including XDR‐TB). RIF‐mono, rifampicin mono‐resistance; MDR‐TB, multidrug‐resistant TB; PHC, primary health care; USD, US dollars.

The overall cost and contributions of cost categories varied considerably by drug resistance profile. Patients infected with pre‐XDR and XDR‐TB strains (grouped together due to smaller numbers) were associated with a mean cost of USD 15 567 (range 1472–87 140), more than 2.5 times that of patients with rifampicin mono‐resistance and MDR‐TB (grouped together due to very similar cost), at USD 6852 (range 260–49 577). Although drug‐use costs were higher in the pre‐XDR/XDR‐TB group, the major cost difference was due to hospitalisation (Figure 2). On average, hospitalisation of these patients was associated with a mean cost greater than USD 9611. However, due to smaller overall numbers, the cost of treating pre‐XDR and XDR‐TB contributed only 24% of the total cost of treatment.

**Figure 3:**
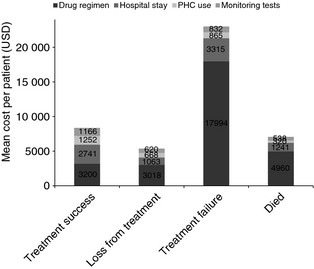
Comparison of mean cost‐by‐cost category between patients with different treatment outcomes. PHC, primary health care; USD, US dollars.

Among successfully treated patients, the mean cost of drugs was higher than the mean hospitalisation cost. For all other outcome categories, hospitalisation was the most significant cost driver (Figure 3).

## Discussion

Our results demonstrate that the cost of RR‐TB treatment under programmatic conditions varies considerably according to resistance profile and treatment outcome. The average cost of treating patients with rifampicin‐resistant TB in Khayelitsha under the decentralised model of care was USD 7916, but ranged from an average of USD 5369 for patients who did not complete treatment to USD 23 006 for those for whom treatment failed. As has been previously reported, treatment failure is particularly associated with second‐line drug resistance at diagnosis 18, 19. As a result, patients with pre‐XDR and XDR‐TB were associated with a much higher average cost at USD 15 567 than patients with rifampicin mono‐resistance or MDR‐TB without second‐line resistance, for whom the average cost of treatment was USD 6852. While the latter cost is similar to the cost of MDR‐TB treatment previously estimated (USD 6772), the cost of treating pre‐XDR and XDR‐TB is substantially lower (USD 26 932) 8. Both these estimates are considerably lower than the 12‐month cost of treatment estimated at USD 17 164, from a programme where all patients were hospitalised 6.

As treatment is only successful for just less than a half of patients, the average cost per treatment success is more than double the average cost per patient at USD 16 214. While treatment failure is particularly costly, successful treatment is more costly on average than the other poor outcomes of death and loss from treatment, predominantly due to the longer duration of treatment. This results in the perverse situation that if treatment outcomes were to improve, overall cost to the health system may actually appear to increase. However, this does not account for the cost to the health system of additional DR‐TB infections acquired from patients lost from or failing treatment. In addition, if through improving treatment outcomes, failure of treatment were to decrease, there is potential for significant cost saving. The introduction of new drugs, such as bedaquiline and delamanid 20, 21, which have the potential to reduce the risk of treatment failure and improve overall outcomes, may improve cost‐effectiveness.

The cost of hospitalisation remains significant, even in a fully decentralised programme such as implemented in Khayelitsha. It is likely that a proportion of patients will require hospitalisation at treatment initiation based on their clinical condition. This proportion may well decrease as patients are diagnosed earlier with the use of rapid diagnostics, such as the Xpert MTB/RIF test 22. However, the main contributor to hospitalisation costs are extended admissions among patients for whom treatment does not work, these are patients with outcomes of treatment failure and death.

The local subacute inpatient facility in Khayelitsha is associated with greater costs than the central TB hospital, but similar costs to smaller, more rural TB hospitals 17. This is predominantly due to economies of scale. However, the benefits of a local facility that is closer to the community and that enables continuity of care between primary care and short‐term inpatient admission are difficult to quantify and have not been considered in this simplified cost analysis. Additionally, admission to a local facility is likely to result in reduced out‐of‐pocket costs for patients and their families. Patients can also be admitted for reasons other than purely medical, such as to assist in medication adherence and for short‐term social problems, and they remain close to the community reducing feelings of isolation. Greater inpatient costs for the subacute facility may have also resulted from the use of MSF staff costs, which have since been aligned to government salary scales, resulting in significantly reduced overall costs. In addition, a significant proportion of the running cost of this facility was due to the mechanical ventilation system installed to provide a safer environment for other patients and staff.

The cost of second‐line drugs remains significant, particularly for patients with pre‐XDR and XDR‐TB. Much of this cost is driven by the routine inclusion of capreomycin in the regimen, in the absence of demonstrated capreomycin susceptibility. High levels of cross‐resistance have been demonstrated in South Africa 23, and it is likely that capreomycin is not efficacious in the regimen for a significant proportion of patients. The recommendation for routine hospitalisation in this group of patients is driven in part by the need for closer clinical monitoring to detect renal toxicity due to capreomycin 24. The introduction of capreomycin susceptibility testing and restriction of capreomycin to only those patients with demonstrated susceptibility are likely to both reduce costs through reduced drug use and hospitalisation in addition to reducing severe adverse events, including death due to renal failure, among patients.

This analysis has several limitations, primarily due to the use of routine data. Missing data on the duration of the intensive phase and length of stay in hospital were imputed for a proportion of patients, and data on changes to treatment regimens throughout treatment were not routinely recorded. We assumed that all patients were treated with regimens according to the guidelines. However, in practice, there are variations to regimens in a minority of patients and regimens are adjusted throughout treatment due to adverse events or the development of further drug resistance. Similarly, data on what tests were actually conducted were also not recorded routinely; some patients will have had more intense clinical and laboratory monitoring compared to the guidelines and others potentially less. There are also inherent limitations in calculating the costs associated with one disease in a setting of integrated service delivery.

Despite these limitations, these data provide estimates of the cost per patient of treatment for RR‐TB using a decentralised model of care in South Africa. The results demonstrate that costs vary considerably by drug resistance profile and by treatment outcome, and can be used to model the cost‐effectiveness of introducing new treatment regimens with potentially improved treatment outcomes.
